# Risk Factors and Outcome Analysis in Rupture of Gravid Uterus: Lessons for Obstetricians

**DOI:** 10.7759/cureus.21890

**Published:** 2022-02-03

**Authors:** Sheeba Marwah, Swati Singh, Neha Bharti, Prashanta K Gupta

**Affiliations:** 1 Obstetrics and Gynaecology, Vardhman Mahavir Medical College & Safdarjung Hospital, New Delhi, IND

**Keywords:** perinatal mortality, maternal mortality, hysterectomy, caesarean section, uterine rupture

## Abstract

Objective

This study was conducted to determine the risk factors and feto-maternal outcomes in uterine rupture at a tertiary care centre, with the goal to assess the delays or gaps in management, in order to avert associated morbidity and mortality.

Material and methods

This study was conducted from June 2018 to May 2020 in Vardhman Mahavir Medical College & Safdarjung Hospital, New Delhi, wherein all women diagnosed with uterine rupture, either at the time of admission or during the course of hospital stay, were included after taking written informed consent. The primary outcome measured was the incidence of uterine rupture, whereas the secondary outcomes assessed were clinical features, risk factors, per-operative findings, management, and feto-maternal outcomes.

Results

The total number of deliveries during the study period was 67005. Out of these, 12985 women underwent LSCS, whereas others delivered vaginally. A total of 61 cases of uterine rupture occurred among them. The majority of these women were unbooked (62.29%), having a gestation age >37 weeks (65.57%). The most common risk factor identified was a history of previous LSCS (91.80%). Around 80.33% of women had rupture of the lower segment of the uterus. Maximum cases were managed by repair with ligation (63.93%), while 26.22% underwent hysterectomies. Bladder injury occurred in 11.48% of women. While most of the women required blood transfusion (93.44%), only three maternal deaths occurred.

Conclusion

Rupture of a gravid uterus can be a lethal surgical catastrophe with potentially grave feto-maternal consequences. Alacrity in diagnosis and referral to a tertiary centre, along with facility-level preparedness to respond to this emergency, apart from optimal care around birth, are critical determinants for feto-maternal survival.

## Introduction

Uterine rupture in pregnancy is an infrequent, but cataclysmic impediment with a high incidence of fetal and maternal morbidity and mortality. It has an incidence of <1% in women with scarred uteri; however, it is extremely rare in the unscarred uterus with an alluded incidence of only 0.006% [1,2]. The occurrence of this clinical entity has steadily mounted in figures over the recent decades [3-5]. Due to changing trends of advanced maternal age at the time of conception, rising number of trans-myometrial surgeries prior to conception, increasing caesarean sections rates, and a higher rate of induction of labour by means of prostaglandins and oxytocin, the number of cases of rupture uterus is rising. However, with enhancement in contemporary obstetric services, cases of uterine rupture following previously unscarred uterus are declining [6]. Uterine rupture classically refers to a complete separation of all the uterine layers and of the overlying visceral peritoneum and is often associated with clinically significant paroxysmal pain, uterine bleeding, fetal distress, and even protrusion or expulsion of the fetus and/or placenta into the abdominal cavity [7], but when the peritoneum is still intact, it is referred to as incomplete rupture.

The rate of uterine rupture is known to increase in patients with advanced maternal age, overdue pregnancy, macrosomia, a shorter interval of deliveries, single-layer uterine closure, multiple previous caesarean deliveries, and trial of labour after caesarean section as well as laparoscopic or abdominal myomectomy or adeno-myomectomy but it can also occur in women with a native, unscarred uterus. The most common risk factor for uterine rupture is uterine scarring from a previous caesarean section. In one review, 52% had previous caesarean scars [8]. An extremely rare case of uterine rupture in the first pregnancy with no risk factors has also been reported [9]. The risk factors in cases of an unscarred uterus may be associated with the weakness of the myometrium due to trauma, congenital anomaly, multiple gestations, and the use of uterotonic drugs.

Previous caesarean scar rupture is frequently diagnosed on the basis of altered fetal heart rate pattern, vaginal bleeding, maternal tachycardia or unusual pain during labour. For rapid and accurate identification of the aetiology of abdominal pain, non-contrast MRI is being increasingly used in pregnant patients in the emergency setting [10]. The complications could be severe including maternal haemorrhage, blood transfusion, hysterectomy, bladder injury, maternal death as well as fetal prematurity, lower Apgar scores and death. The promptness with which the patient is managed, availability of blood transfusion, competent surgical intervention and adequate anaesthesia determines maternal outcome from rupture uterus. The type of surgical intervention on the uterus is dependent on the type and extent of the rupture, hemodynamic status of the mother, desire for future fertility, presence of gross infection and experience level of the surgeon. There could be subtotal abdominal hysterectomy, uterine repair with or without tubal ligation. Uterine repair should be reserved for women who have a low transverse rupture, no extension of the tears to broad ligaments, cervix or vagina, easily controllable haemorrhage, good general condition, desire for future childbearing and no evidence of gross infection. Hysterectomy is appropriate for those without the above intraoperative findings. The poorer outcomes may result from delayed identification and management because of the unexpectedness and rareness.

Our hospital is one of the largest referral centres in the country under the central government and receives a high number of referrals from the peripheries. Our aim is to study the risks and feto-maternal outcomes of uterine rupture in our centre so that we can prevent morbidity and mortality. Delay in management places both mother and child at significant risk. All gynaecologists need to be equipped to deliver early and prompt diagnosis and treatment.

## Materials and methods

After taking ethical clearance with the Institutional Ethics Committee of Vardhaman Mahavir Medical College & Safdarjung Hospital with the approval no. S.No. IEC/VMMC/SJH/ Project/2020-07/CC-04, this retrospective study was conducted in the department of obstetrics and gynaecology of VMMC & Safdarjung hospital from June 2018 to May 2020, wherein all women diagnosed with uterine rupture either at the time of admission or during hospital stay were included. The patient's details were extracted from case records and data entered in pre-assigned case proforma. The primary outcome measured was the incidence of uterine rupture, whereas secondary outcomes assessed were risk factors, per-operative findings, management and fetomaternal outcomes.

## Results

During the study duration, 61 women were treated for ruptured uterus in the institute. The total number of deliveries including caesarean section during the same period was 67005. Thus, the incidence of ruptured uterus came to 0.1%. Around half of the women were in the age group of 25-29 years (mean age 27 years) and overweight (52.46%) with a mean BMI in the range of 25kg/m2. Most of the women were multigravida (96.72%) and around two-thirds (62.3%) of women were unbooked with no prior antenatal visits presenting at term. Notably, two women who were primigravida patients with a ruptured uterus were both unbooked on presenting with obstructed labour on admission. Amongst them, one woman had hand prolapse whilst the other gave a history of treatment from a traditional birth attended and experienced prolonged leaking per vaginum. Also, it was observed that the incidence of ruptured uterus increased with increasing gestational age (Table 1).

**Table 1:** Sociodemographic and obstetric characteristics of the study population

Demographic and obstetric characteristics		Number of patients	Percentage of patients
Age	<20 years	3	4.92
	20-24 years	10	16.39
	25-29 years	29	47.54
	30-34 years	16	26.23
	35-39 years	3	4.92
Parity	G1	2	3.28
	G2-G4	44	72.13
	>G4	15	24.59
Body mass index	15-18.9 Kg/m2	2	3.28
	19-22.9 Kg/m2	14	22.95
	23-26.9 Kg/m2	32	52.46
	27-29.9 Kg/m2	13	21.31
	>30 Kg/m2		0.00
Antenatal care	Unbooked	38	62.29
	Single visit	14	22.95
	Two and >two(other hospital)	7	11.48
	Booked	2	3.28
Gestational age	<22weeks	1	1.64
	22-28 weeks	1	1.64
	29-34 weeks	2	3.28
	33-37 weeks	17	27.87
	>37 weeks	40	65.57

The classical signs of uterine rupture are fetal distress with a non-reassuring fetal heart rate seen in two-thirds of patients. Around 49.1% of patients on examination had abnormal uterine contour with absent fetal heart rate. Others presented with either antepartum haemorrhage or postpartum haemorrhage, sometimes with massive haemorrhage, leading to shock. One patient was brought dead to gynaecology casualty with abnormal uterine contour (Table 2).

**Table 2:** Clinical picture on diagnosis

Clinical Picture	Number of patients	Percentage of patients
Non reassuring fetal heart rate/ Absent fetal movement	42	68.8%
Abnormal uterine contour with absent fetal heart rate	30	49.1%
Antepartum haemorrhage	21	34.4%
Postpartum haemorrhage	20	32.7%
Shock	5	8.1%
Brought dead with rupture	1	1.6%

Most of the women in the study had spontaneous onset of labour (63.39%) with a history of having at least one previous LSCS (70.49%). Unexpectedly, 8.2% of women presented with a ruptured uterus despite no previous scarring. Nearly 50% of women had an inter-delivery interval >18 months. Two-thirds of women had a labour duration of fewer than 12 hours, with one woman having previous three scars progressing to uterine rupture immediately as labour commenced (1.5 hours). Only one patient with two previous LSCS presented with a ruptured uterus at 18 weeks gestation (Table 3).

**Table 3:** Risk factors of ruptured uterus in the study population LSCS; lower segment cesarean section H/O: history of; TOLAC: trial of labour after cesarean

Risk factors for rupture uterus		Sub Category	Number of patients	Percentage of patients
Labour	Spontaneous	-	39	63.93
	Induced	Gel	12.00	19.67
		Oxytocin	8.00	13.11
		Misoprostol	2.00	3.28
Scarred/unscarred uterus	Unscarred uterus	-	5	8.20
	Previous surgery	Previous 1 LSCS	43	70.49
		Previous 2 LSCS	12	19.67
		Previous 3 LSCS	1	1.64
	Previous preterm LSCS	-	9	14.75
	H/O of wound infection	-	7	11.48
	H/O dilatation and curettage	-	21	34.43
	H/O myomectomy	-	3	4.92
	TOLAC	-	4	6.56
Inter-delivery Interval	<9 months	-	4	6.56
	9-18 months	-	21	34.42
	>18 months	-	31	50.82
Duration of Labor	<12 hours		43	70.49
	12-24hours		15	24.59
	>24hours		3	4.92

**Figure 1:**
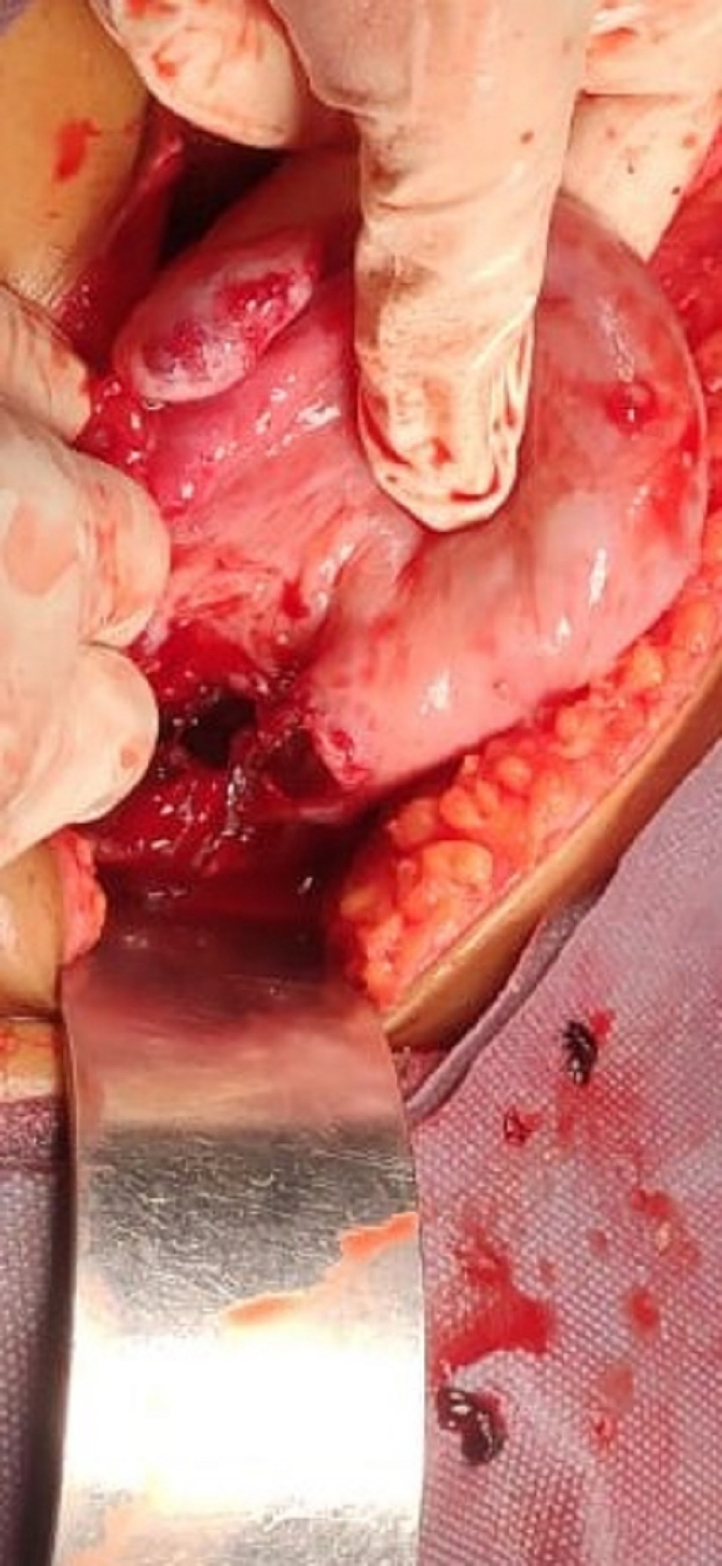
Rupture uterus with site of rupture at previous scar site

**Figure 2:**
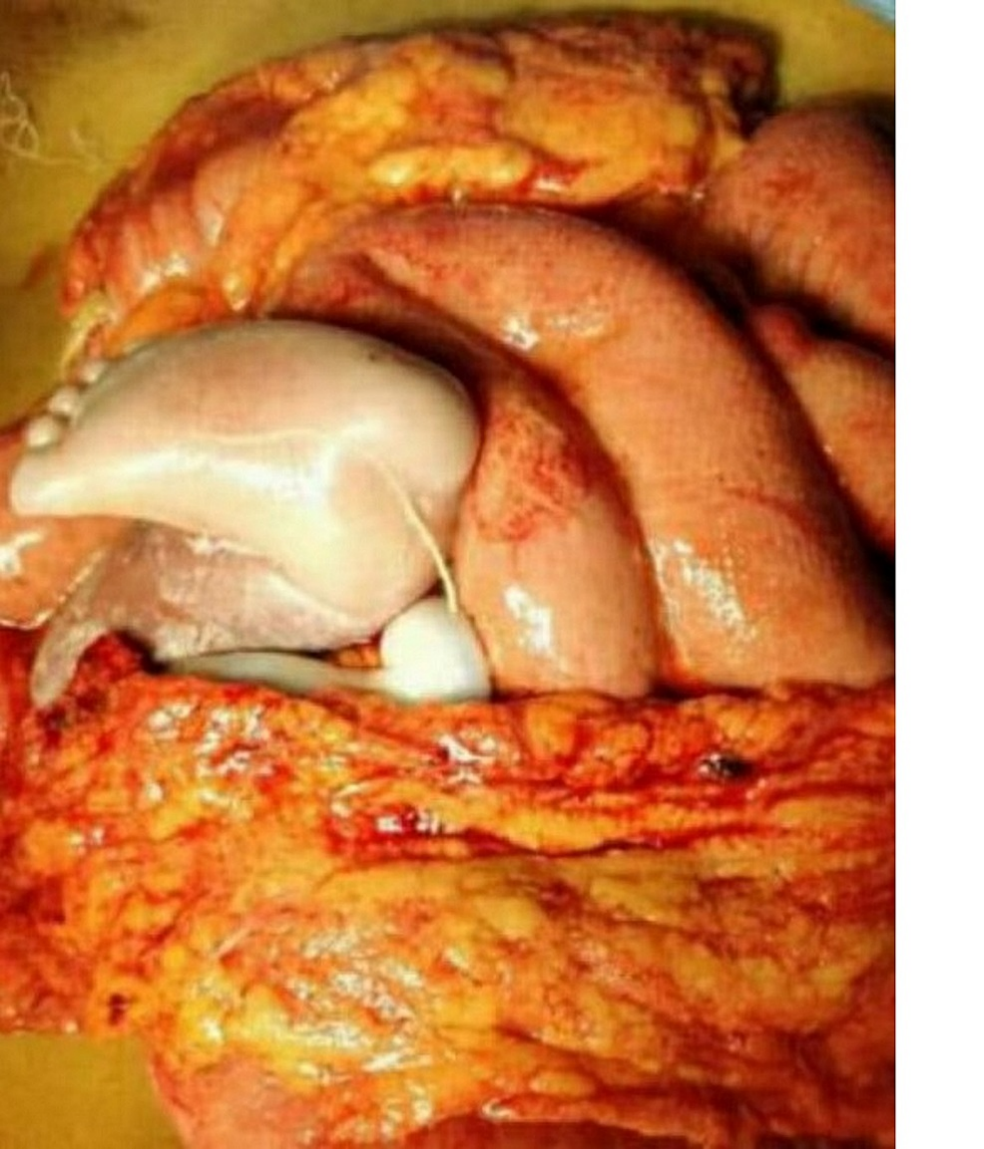
Rupture uterus with baby in intraperitoneal cavity

Almost half of women with a ruptured uterus were diagnosed mostly intraoperatively (49.1%) when taken for emergency caesarean in view of impending scar rupture (Figures 1-2). Of all the patients who were taken for TOLAC, four patients had ruptured uteri. Two women who underwent trials of labour after cesarean (TOLAC) had ruptured uterus diagnosed during the postpartum period. Intraoperatively, it was found that most cases of uterine rupture (86.89%) were complete ruptures involving the lower segment. Complications such as extension and hematoma formation were seen in 19.67% and 6.56% patients respectively. Around 11% of patients had extensions requiring bladder repair. The repair of the rupture site with ligation was done for most of the patients (63.93%) but for 26.23% of patients, the rupture was beyond repair and hysterectomy was performed. Two women had massive PPH requiring internal artery ligation (Table 4).

**Table 4:** Per Op Findings / Interventions of study participants

Per op findings / Interventions	Per op findings / Interventions	Number of patients	Percentage of patients
Type of Uterine Rupture	Complete Rupture	53	86.89
	Incomplete Rupture	8	14.75
Site of uterine rupture	Lower segment	49	80.33
	Upper segment	4	6.56
Complication	Extension	12	19.67
	Hematoma	4	6.56
Surgery	Repair	6	9.84
	Repair with ligation	39	63.93
	Subtotal hysterectomy	5	8.20
	Hysterectomy	11	18.03
	Bladder repair	7	11.48
	Internal iliac ligation	2	3.28

Rupture uterus is an acute emergency that poses significant maternal and neonatal morbidity and mortality. Almost all (93.44%) women had blood transfusions due to massive blood loss, both intrapartum and postpartum. Despite our best efforts, three women succumbed to severe clinical conditions and had maternal mortality. Only 14% of babies were live born and almost all (eight out of nine) liveborn required NICU admission (Table 5).

**Table 5:** Fetomaternal outcomes

Feto maternal outcome	Number of patients	Percentage of patients
Need of blood transfusion	57	93.44
Postpartum haemorrhage	13	21.31
Intensive care unit admission	8	13.11
Mortality	3	4.91
Live born	9	14.75
Stillborn	52	85.25
Admission in neonatal intensive care unit, Apgar in 1 minute <7, Apgar in 5 minutes <7	8, 7, 4	13.11, 11.47, 6.55

## Discussion

The incidence of uterine rupture in the present study was ultimately 0.1%, a very low rate contrary to that deduced in a recent meta-analysis [1]. This can be attributed to the constant efforts in promoting readiness of the working doctors in the department to correctly delineate candidates of TOLAC and recognize early signs of impending rupture and preventing the morbidity associated with uterine rupture. This difference may be attributed to the study being single centred study. Also, the institute is a tertiary care referral centre with set standard operating procedures for the management of pregnancy with previous uterine scarring, and also instituted judicious obstetric interventions, good antenatal care, early birth preparedness and complication readiness plans. This highlights the importance of region-wise healthcare service provision to promote wider utilization of accessible health services in this cohort of women. Also, promotion of smaller family sizes, adequate spacing between conceptions, and judiciously identifying the indication of caesarean helps to achieve the same.

The majority of women with uterine rupture were unbooked. Similar results were found in other studies [11-13]. So the need of the hour is that women should have regular antenatal visits so high-risk factors could be identified and time, mode and place of delivery could be planned. Also, there should be timely referrals of patients with signs of impending rupture to tertiary care facilities. Most of the women were in the age group 25-29 years. Similar ﬁndings were found in other studies also as it is the age of maximum fertility [11-14]. In addition, multiparity was another risk factor noted in the study. In obese women, there was an increased number of cases of uterine rupture as there are increased chances of labour dystocia and difficult labour monitoring.

The onset and rupture areas varied from patient to patient; however; most of the uterine ruptures occurred after 37 weeks of gestational age and were located at previous scars. The occurrence after 37 weeks may be associated with uterine enlargement in the third trimesters or subclinical uterine contractions. You et al. and Bereka et al. found maximum uterine rupture >30 weeks and >37 weeks respectively [15,16]. Only one patient presented with a ruptured uterus at 18 weeks with a history of previous two LSCSs. Contrary to our belief, it was seen that in women with spontaneous onset of labour there were more cases of rupture uterus as compared to induced labour. So labour monitoring is very important in order to diagnose signs of impending rupture and intervene in a timely manner.

Signs and symptoms of uterine rupture largely depend on the period of gestation, the site and the extent of the uterine defect. Spontaneous or traumatic rupture of the uterus is more catastrophic than uterine rupture at the site of previous uterine scarring because of relatively reduced vascularity at the previous scar site. Classical signs of uterine rupture are fetal distress with a non-reassuring fetal heart rate seen in two-thirds of patients. Around 50% of patients on examination had abnormal uterine contour with absent fetal heart rate. Others presented with either antepartum haemorrhage or postpartum haemorrhage, sometimes with massive haemorrhage leading to shock. One patient was brought dead to gynaecology casualty with abnormal uterine contour.

During the period of study, we had 1265 TOLAC amongst which four women had uterine ruptures. Thus, the incidence of rupture uterus during TOLAC in our institute was 0.31% and the literature reported rupture rate in TOLAC was about 0.78% [17]. There were 245 successful cases of VBAC in our institution during the study period. However, the other patients had failed TOLAC because of indications other than rupture such as NRFHT caused by other aetiologies. The most common risk factor of uterine rupture is previous caesarean sections. It was seen that in women with short inter-conceptional periods there were fewer cases of uterine rupture as compared to patients with inter delivery interval >18months which is, in contrast, to study conducted by You et al. [15] as patients with short inter conceptional period did not attempt a trial of labour and were directly taken for caesarean section. TOLAC in cases with short pregnancy interval is associated with an increased risk of rupture and hence result in major morbidity and blood transfusion similar to a study by Kaczmarczyk et al [18].

Other risk factors for uterine rupture are previous preterm LSCSs, previous history of wound sepsis, history of myomectomy and short inter-conception period and patient who underwent dilatation and curettage. A high index of suspicion should be kept in patients with a scarred uterus. In patients with impending rupture, timely referral and preventing delays in the golden hour is very important. With increasing caesarean section rates good surgical techniques need to be inculcated in budding gynaecologists along with sterile surgical practices to prevent complications. Triaging of patients once they reach the referral centre should take place so that the patient receives adequate management to prevent unnecessary caesarean. Resources should be increased and the provision of back referral of low-risk pregnancies be present so the right treatment to the right patient could be given. It should be ensured to correct anaemia and maintain hygienic practices, which would prevent infections and promote proper wound healing. All patients undergoing caesareans should be counselled regarding family planning services to maintain adequate interpregnancy interval which helps in proper wound healing and also to promote TOLAC in the next pregnancy. 

Two-thirds of cases underwent uterine repair with ligation. The procedure which is the shortest in duration, does not aggravate the patients' state of shock, and will get the patient off the operating table in the best possible condition, is the best procedure for a ruptured uterus [12]. Blood transfusions were required in almost all cases.

A uterine rupture is an abrupt event that is life-threatening to both mother and fetus and may not be preceded by uterine contractions. Both scarred and unscarred ruptures are concentrated after 30 weeks of gestation. The incidence of rupture in unscarred uteri occurred in later gestation, however, these cases had remarkably morbid outcomes as well. Atypical low abdominal pain not only in women with uterine scars and short intervals between prior surgery and conception but also the individuals without uterine scars from the third trimester till postpartum should be of concern to obstetricians. Though there was no clinical reliable prediction or prevention for uterine rupture, obstetrician awareness and vigilance and timely management could decrease maternal and neonatal morbidity.

## Conclusions

The rupture of a gravid uterus is a potentially lethal surgical catastrophe with grave feto-maternal outcomes. Alertness in diagnosis, referral to a tertiary centre and facility-level preparedness to respond to this emergency, apart from optimal care around birth, are critical determinants for feto-maternal survival.

The impact of uterine rupture highly affects the life of the fetus, resulting in a spectrum of low Apgar score to stillbirth; and mothers are affected by the problem, ranging from immediate complications to long-lasting impacts, such as loss of fertility. To improve outcomes, coordinated effort must be made in health institutions at the community level and policymakers need to give special emphasis to rural areas in particular when considering the enhancement of access and utilization of medical services.
